# ICMA: an integrated cardiac modeling and analysis platform

**DOI:** 10.1093/bioinformatics/btu809

**Published:** 2014-12-06

**Authors:** Jagir R. Hussan, Peter J. Hunter, Patrick A. Gladding, Neil Greenberg, Richard Christie, Alan Wu, Hugh Sorby, James D. Thomas

**Affiliations:** ^1^Auckland Bioengineering Institute, University of Auckland, Auckland, New Zealand, ^2^Waitemata District Health Board, North Shore Hospital, Auckland 0622, New Zealand, ^3^National Space Biomedical Research Institute, Houston, TX 77030-1402, USA, ^4^Cleveland Clinic Foundation, Cleveland, OH 44195, USA and ^5^Feinberg School of Medicine, Northwestern University, Chicago, IL 60611, USA

## Abstract

**Summary:** ICMA, a software framework to create 3D finite element models of the left ventricle from cardiac ultrasound or magnetic resonance imaging (MRI) data, has been made available as an open-source code. The framework is hardware vendor independent and uses speckle tracking (endocardial border detection) on ultrasound (MRI) imaging data in the form of DICOM. Standard American Heart Association segment-based strain analysis can be performed using a browser-based interface. The speckle tracking, border detection and model fitting methods are implemented in C++ using open-source tools. They are wrapped as web services and orchestrated via a JBOSS-based application server.

**Availability and implementation:** The source code for ICMA is freely available under MPL 1.1 or GPL 2.0 or LGPL 2.1 license at https://github.com/ABI-Software-Laboratory/ICMA and a standalone virtual machine at http://goo.gl/M4lJKH for download.

**Contact:** r.jagir@auckland.ac.nz

**Supplementary information:** Supplementary materials are available at *Bioinformatics* online.

## 1 Introduction

Cardiac echocardiography is a widely used modality to assess wall motion due to its non-invasiveness and comparatively lower setup costs. Most ultrasound vendors provide proprietary software to analyze the echocardiogram, for instance, GE’s EchoPac and Phillips’s QLAB. The software enables cardiologists to determine clinically relevant parameters such as ejection-fraction, regional wall strain etc. However, these software components are expensive, tied to the vendor hardware and not open-source.

Clinical reports generated by the software vary across vendors for the same patient data and therefore there is a lack of standardization across the vendors. This makes it difficult to perform multi-site, population-based studies where data are collected from hardware developed by a multitude of vendors.

We developed a model guided speckle tracking method that tracks left ventricular wall motion in cardiac ultrasound images. The motion data are then used to fit a finite element model (FEM) to reconstruct the ventricular geometry. Motion information from one or more ultrasound views can be combined to improve the reconstructed geometry. These modules are independent of each other and wall motion data gathered from other modalities like MRI can also be used. Diagnostic parameters such as regional strain, ejection-fraction, etc. are determined from the model that represents the ventricular geometry.

Using a browser-based graphical user interface, with a workflow similar to existing software, the user can guide the geometry reconstruction process by choosing the images to be used, providing landmarks at the beginning and end R-wave frames (the framework automatically tracks them over the cardiac cycle) for each of these images and verifying the tracked motion. Administrative tasks such as adding and removing subjects, removing models created by users, etc. are also invoked through browser-based GUIs.

The framework provides complete regional strain analysis metrics as offered by commercially available packages. There are no restrictions/limitations on using these available features. Modifications and subsequent distributions are subject to the provisions of Mozilla Public License Version 1.1 (the ‘MPL’) or GNU General Public License Version 2 or later (the ‘GPL’), or the GNU Lesser General Public License Version 2.1 or later (the ‘LGPL’).

## 2 Implementation details

**Fig. 1.**
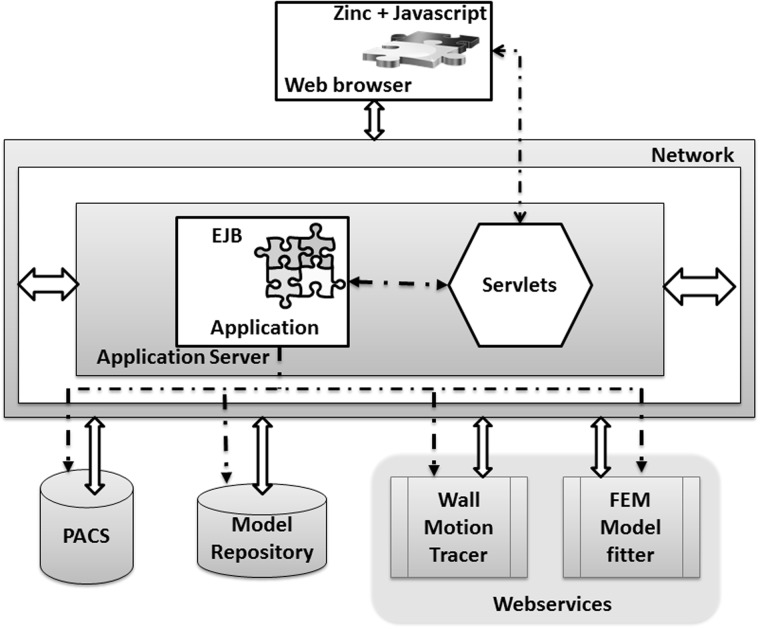
Schematic of ICMA module integration. Block arrows represent data exchange, dash arrows represent control flow

The framework consists of loosely coupled software modules. A schematic of module integration is shown in Figure 1. DICOM data are stored in PACS that exposes WADO; currently DCM4CHEE (www.dcm4che.org) with a MySQL database (www.mysql.com) is used. DICOM data are preprocessed using ffmpeg (www.ffmpeg.org) to generate web optimized movies and images. These files along with metadata from the DICOM header are stored in a MySQL database (model repository).

To perform quantitative analysis of the imaging data, the user has to reconstruct the ventricular geometry by selecting images of interest. For each of the selected images, view plane information (short axis, apical long axis, four chamber, etc.), cardiac cycle of interest and geometric landmarks that aid in tracking the wall motion should be provided. A browser-based user interface provides various controls to select PACS data, to perform geometry reconstruction and to render them. The user interface is developed using HTML and JavaScript (dojotoolkit.org, jquery.com). The 3D mesh is rendered using zinc-browser plugin (www.cmiss.org/cmgui/zinc) and must be installed on the user’s computer.

Compute intensive processes are hosted on the server and the user interface interacts with the server via servlets. A J2EE-based web application orchestrates the model generation and rendering pipeline. It has developed on JBOSS AS 7.1.1 webserver (jbossas.jboss.org/). The models are stored in the model repository along with the study metadata, movies and images.

The speckle tracking module implements the speckle tracking method discussed in (Leitman *et al.*, 2004) along with an adaptive left ventricular model (Young *et al.*, 2000) to robustly follow the wall motion in noisy images and to speed up the process by restricting the tracking process to regions of interest. Image processing was done using ITK 4.2 toolkit (itk.org). DICOM processing was aided by gdcm (sourceforge.net/projects/gdcm/).

The model fitting module is based on the framework proposed by Young *et al.* (1994). Mesh representation and manipulation was done using OpenCMISS-Zinc library (opencmiss.org/zinc), fit optimization was performed using gmm library (home.gna.org/getfem/). Alglib (www.alglib.net) was used to determine the radial basis-based displacement field and smoothing of wall motion. 

Each of these modules are wrapped as a web service and hosted on a JBOSS AS 7.1.1 instance. Note that all these modules have been developed on readily available open-source tools.

## 3 Discussion

ICMA is a software framework for quantitatively assessing wall motion from cardiac ultrasound and MRI data. The framework processes DICOM outputs from the imaging hardware and is therefore independent of the hardware vendor. 

We compared the quantitative metrics produced by this framework with some of the widely used proprietary software using Bland–Altman analysis and the repeatability coefficient (RPC) for the framework was 5.3, with the intraobserver RPC being 4.5. The best performing proprietary software had an RPC of 3.0 (see Supplementary information for details).

Unlike proprietary software, the framework is open-source and the wall motion tracking methods can be readily accessed, analyzed and modified. Also, different methods for performing wall motion tracking can be easily plugged into the framework.

The ventricular geometry is available as a finite element mesh and this enables

1. Easy development of metrics.
2. Coupling of the imaging data to computer simulations (Sermesant *et al.*, 2012).
3. Integrating a multitude of clinical data to deliver patient specific care (Gladding *et al.*, 2013).

A key feature of ICMA is that the user interface is browser based and allows data to be accessed across a network.

With advances in medical device technologies a variety of portable imaging devices are being developed. Semantic cohesiveness of assessment metrics becomes critical for successful and large scale deployment of such devices. Our primary motivation to release ICMA as an open-source technology is with the hope that it would be adopted by the community to

1. Develop new modules to integrate portable devices.
2. Develop new modules to use the FEM models.
3. Improve upon the existing modules.
4. Incorporate other visualization/analysis systems.
5. Provide a reference standard for metrics.

Further, to the best of our knowledge we are not aware of any open-source or freely available cardiac-ultrasound strain analysis software.

 In USA, annual imaging costs alone exceed $100 billion (Iglehart, 2006) and cardiovascular imaging accounts for 29% of all medical imaging (Levin *et al.*, 2005). About one-third of all annual medical imaging costs worldwide can be attributed to cardiovascular imaging (Picano, 2005). This may be partly attributed to proprietary software products and their designed incompatibility with other software and hardware vendors. We believe that an open-source community-led effort to develop software for such applications can reduce these costs and ICMA presents a model implementation that can be improved upon.

## Funding

This work was funded by National Space Biomedical Research Institute, USA (CA02203).

*Conflict of Interest:* none declared.

## Supplementary Material

Supplementary Data
