# Supplementary material for: ICMA: an integrated cardiac modeling and analysis platform
Source: Bioinformatics. 2014 Dec 6;31(8):1331–3. doi: 10.1093/bioinformatics/btu809 (PMC4393521; doi:10.1093/bioinformatics/btu809)
Supplement: Supplementary Data [file supp_31_8_1331__index.html]

ICMA: An integrated Cardiac modeling and Analysis platform — ICMA: an integrated cardiac modeling and analysis platform — ICMA: an integrated cardiac modeling and analysis platform — Supplementary Data 

# ICMA: an integrated cardiac modeling and analysis platform

## Supplementary Data

files

**Files in this Data Supplement:**

- Supplementary Data - docx file
