# Supplementary material for: ICMA: an integrated cardiac modeling and analysis platform
Source: Bioinformatics. 2014 Dec 6;31(8):1331–3. doi: 10.1093/bioinformatics/btu809 (PMC4393521; doi:10.1093/bioinformatics/btu809)
Supplement: Supplementary Data [file supp_btu809_ICMA_supplementalr1.docx]

ICMA: An integrated Cardiac modeling and Analysis platform

Jagir R. Hussan^1^, Peter J. Hunter^1^, Patrick A. Gladding^2^, Neil Greenberg^3,5^, Richard Christie^1^, Alan Wu^1^, Hugh Sorby^1^, and James D. Thomas^3,4^

**Supplementary information**

Address for correspondence:

Auckland Bioengineering Institute

Level 6, 70, UniServices House, Symonds Street

Auckland 1010, New Zealand

Phone: +64 9 373 7599 ext. 85360

Email: r.jagir@auckland.ac.nz

Affiliations:

^1^Auckland Bioengineering Institute, University of Auckland, Auckland, New Zealand.

^2^Waitemata District Health Board, North Shore Hospital, Private Bag 93503, Auckland 0622, New Zealand.

^3^National Space Biomedical Research Institute, 6500 Main Street, Suite 910, Houston, TX 77030-1402, USA

^4^Feinberg School of Medicine, Northwestern University, 676 N Saint Clair Chicago IL 60611, USA

^5^Cleveland Clinic Foundation, 9500 Euclid Avenue, Cleveland, OH 44195, USA

**INTRODUCTION**

We compared ICMA implementation of speckle based motion tracking with existing proprietary software to evaluate its performance. First, we present these results and our observations.

**COMPARISON METRICS**

The speckle tracking based strain analysis methods were compared based on the following metrics.

1. Single observer reproducibility - determine whether the method can reproduce the results when the strain analysis is repeated by the same observer
2. Inter-observer reproducibility - determine whether the method can reproduce the results when the strain analysis is repeated by a different observer
3. Inter-method reproducibility - determine whether the results produced by the strain analysis methods quantitatively match when repeated by the same observer

**STRAIN ANALYSIS METRIC**

For each long axis view plane (Four Chamber, Two Chamber, Apical Long Axis view), strain analysis was performed and the regional strain curves over a cardiac cycle (R-wave to R-wave) were determined.

Strain curves that are not physiological were eliminated and the average of the remaining strain curves was determined. The maximum strain value (magnitude) of this average strain curve was calculated and called the *peak global longitudinal strain*. This metric was used to compare strain analysis experiments.

**DATASET**

Imaging data from subjects with Hypertrophic Cardiac Myopathy, Ischemic Cardiac Myopathy, Non-ischemic Cardiac Myopathy and Normal volunteers were used. There were two subjects per phenotype and three long axis views (Four chamber, Two Chamber and Apical Long Axis) per subject. The imaging hardware varied (by vendor and version) across the subjects and was primarily acquired from GE or Phillips machines.

**RESULTS**

The images were read and analyzed using GE EchoPac, Phillips VVI and ICMA strain analysis module, independently by two cardiac consultants. Each image was analyzed twice (not in succession) and the peak global longitudinal strain for the image+method+operator combination was recorded.

|  | EchoPac | VVI | ICMA |
| --- | --- | --- | --- |
| EchoPac | 3 | 4.4 | 6.1 |
| VVI | 4.4 | 4.8 | 6.2 |
| ICMA | 6.1 | 6.2 | 5.3 |

Table 1: Repeatability coefficient determined using Bland-Altman analysis for imaging data using three strain analysis methods. The repeatability coefficient for the two analysts was 4.5

This data was then analyzed using Bland-Altman analysis and the repeatability coefficient (RPC) was determined (Table 1).

The repeatability coefficient measures the absolute difference between any two future measurements made by that particular observer/method on a particular subject/unit. This difference is estimated to be no greater than the RPC value on 95% of occasions. It is important to note that the repeatability of another observer may be different, because of differences in the training and ability of observers. The repeatability coefficient for the two cardiologists was 4.5.

The results indicate our implementation has consistently higher difference compared to the proprietary methods. This is due to the additional layer of template matching that the proprietary software employ. Both software, determine landmarks based on the apex and base plane information provided by the user. This eliminates errors that are introduced by small differences in the position of the landmarks when the experiment is repeated.

Also, the software automatically determines the end-systole frame and the base plane position which contributes to better reproducibility. To validate this, we repeated the tracking method with the same landmark data to check if the method has some inherent noise; however, the results were identical in each run.

We also ran experiments with variations to the landmarks by adding a small random number (±7 pixels) and found that our method was sensitive to the variation but with a much lower RPC (average of 0.2 over 100 repeats). However, small changes in the end-diastole frame (±5) lead to larger RPC (average of 1.7 over 100 repeats).
